# Practical guidelines for validation of supervised machine learning models in accelerometer‐based animal behaviour classification

**DOI:** 10.1111/1365-2656.70054

**Published:** 2025-05-19

**Authors:** Oakleigh Wilson, David Schoeman, Andrew Bradley, Christofer Clemente

**Affiliations:** ^1^ University of the Sunshine Coast Sippy Downs Queensland Australia

**Keywords:** biologging, cross‐validation, IMU, movement ecology, overfitting

## Abstract

1. Supervised machine learning has been used to detect fine‐scale animal behaviour from accelerometer data, but a standardised protocol for implementing this workflow is currently lacking. As the application of machine learning to ecological problems expands, it is essential to establish technical protocols and validation standards that align with those in other ‘big data’ fields.
2. Overfitting is a prevalent and often misunderstood challenge in machine learning. Overfit models overly adapt to the training data to memorise specific instances rather than to discern the underlying signal. Associated results can indicate high performance on the training set, yet these models are unlikely to generalise to new data. Overfitting can be detected through rigorous validation using independent test sets.
3. Our systematic review of 119 studies using accelerometer‐based supervised machine learning to classify animal behaviour reveals that 79% (94 papers) did not validate their models sufficiently well to robustly identify potential overfitting. Although this does not inherently imply that these models *are* overfit, the absence of independent test sets limits the interpretability of their results.
4. To address these challenges, we provide a theoretical overview of overfitting in the context of animal accelerometry and propose guidelines for optimal validation techniques. Our aim is to equip ecologists with the tools necessary to adapt general machine learning validation theory to the specific requirements of biologging, facilitating reliable overfitting detection and advancing the field.

## Graphical abstract

Overfitting is a major challenge in supervised machine learning and requires thorough validation to detect. A review of the animal accelerometer‐based behaviour classification literature revealed most studies did not adequately validate for overfitting. Guidelines are proposed to help ecologists ensure robust validation for supervised machine learning models for time‐series data.

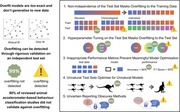

## 1 INTRODUCTION

### 1.1 The golden age of machine learning in biologging

**FIGURE 1:**
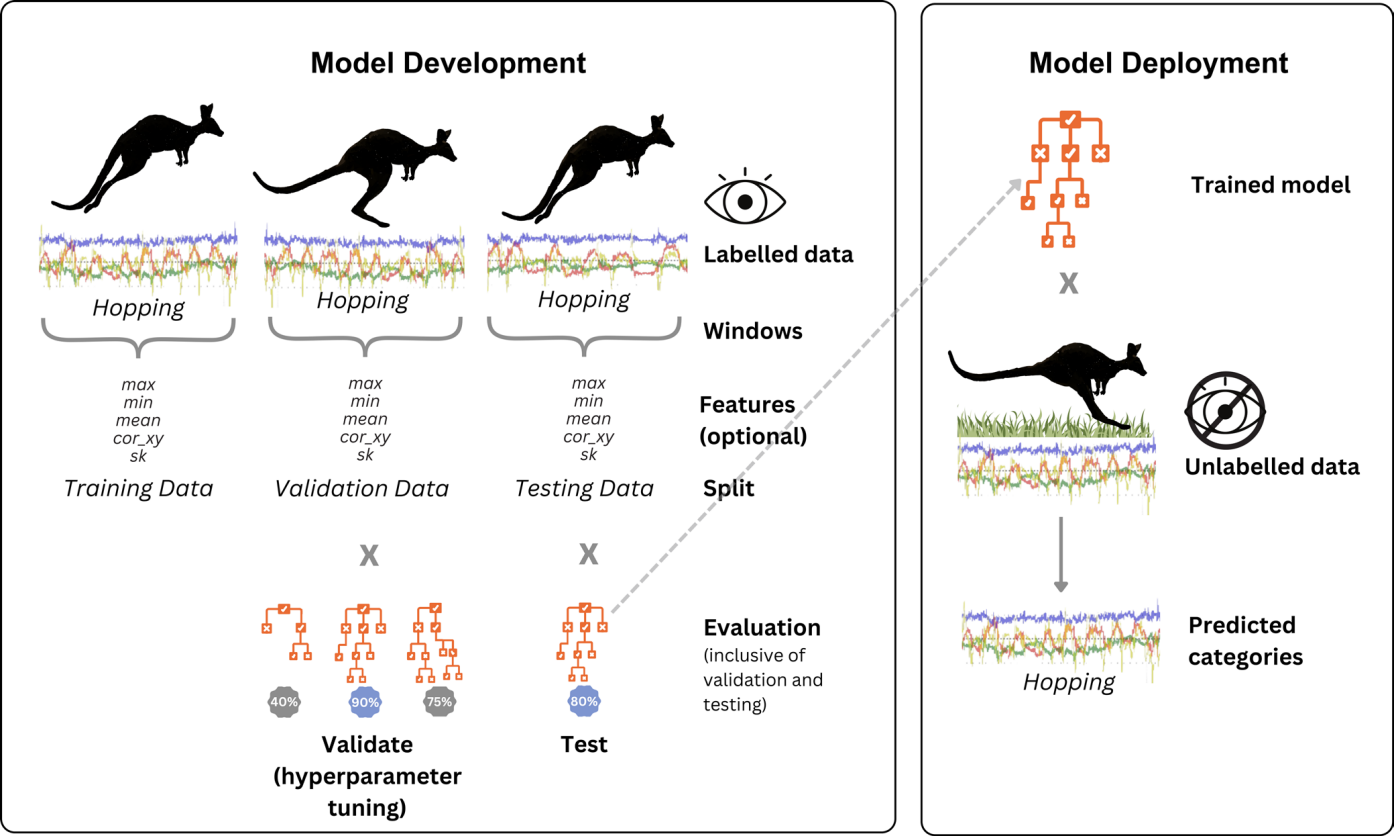
Overview of the stages involved in developing a supervised machine learning model for animal accelerometry. In the model development phase, a machine learning architecture is trained to recognise patterns in labelled training data. The training data are made up of ‘windows’ (discrete units of time‐series data). While deep learning systems autonomously generate features from raw data, traditional machine learning approaches require feature extraction, where summary statistics (e.g. mean, maximum, minimum) are computed for each window. These features reduce the high dimensionality of the raw data, facilitating easier classification for smaller models. Each window is associated with behaviour labels, such as ‘sleeping’, ‘running’ or ‘feeding’. Evaluation comprises two stages. Model hyperparameters are tuned using a validation set composed of independent windows. The final model's performance is then calculated on a separate test set, which assesses the model's ability to classify new, unseen data not included during training. This process allows for the assessment of performance in novel scenarios. Behavioural labels can then be predicted for unseen data using the trained model.

Biologging, particularly animal‐borne accelerometry, has enabled unprecedented insights into the secret lives of wild animals, allowing biologists to track activity levels (Brown et al., 2013), energy expenditure (Wilson et al., 2020) and even fine‐scale behaviours (Brown et al., 2013; Sur et al., 2023) across hundreds of species. Accelerometers record sequences of instantaneous acceleration which can be linked to corresponding causal behaviours. Machine learning (ML) models can then be trained to identify similar patterns in new data from unobserved individuals, for which behaviours are not known (Figure 1; Brown et al., 2013; Sur et al., 2023). ML in this field can be broadly classified into supervised learning, which relies on labelled examples to train the ML model, unsupervised learning, which operates without labelled examples, and semi‐supervised learning which combines elements of both techniques. Here, we analyse validation methods for supervised models (Figure 1), omitting semi‐supervised and unsupervised models as they typically do not ‘validate’ in the traditional sense and are not yet as popular as supervised models (Sur et al., 2023).

ML is rapidly enhancing the scope of biological research, with an exponential acceleration in ML utilisation across fields (Greener et al., 2022; Jones, 2019). Despite this technology's increased accessibility, it remains a technical specialisation that requires correct application to avoid misleading results (Greener et al., 2022; Jones, 2019; Quinn et al., 2021). Biological fields with a history of ‘big‐data’ computation, such as genomics and bioinformatics, have addressed the need for standardised protocols and reporting guidelines, with the publication of discipline‐specific introductions to ML (Greener et al., 2022; Jones, 2019) and the development of standardised reporting checklists (Walsh et al., 2021). More traditional branches of ecology, however, have yet to adopt this level of ML training and standardisation, with ecologists often independently learning to navigate technical terminology (jargon) and critical design choices without formal training in ML theory or practice (Campbell et al., 2013; McClintock et al., 2014). Advocates for the development of user‐accessible ML protocols for behaviour recognition abound (Ferdinandy et al., 2020; Garde et al., 2022; Yu et al., 2023), but published efforts have focused mainly on hardware and sampling—for example, device positioning (Garde et al., 2022; Gleiss et al., 2011; Kölzsch et al., 2016), sampling frequency (Hounslow et al., 2019; Yu et al., 2023) and window length (Putra & Vesilo, 2017)—with focus on theoretical implementation emerging more recently (Ferdinandy et al., 2020). Specifically, a unified method for model verification has yet to be identified within the animal accelerometery research community.

Before progress can be made on developing more powerful models, it is critical to determine how best these should be validated. Validation is the process of predicting model performance onto an unseen portion of data and assessing how well the model performed. Validation is the cornerstone of model development as it guides model optimisation and enables us to distinguish high‐performing models from low‐performing models (Cawley & Talbot, 2010). Without robust validation, we do not know whether our model effectively generalises to new data or is hyperspecific to the training data. The importance of rigorous validation in animal accelerometry has been demonstrated experimentally (e.g. Aulsebrook et al., 2024; Ferdinandy et al., 2020), but here we aim to provide a theoretical foundation for researchers to develop a deeper understanding of how to identify and implement rigorous validation in animal accelerometry.

### 1.2 Leakage and overfitting

**FIGURE 2:**
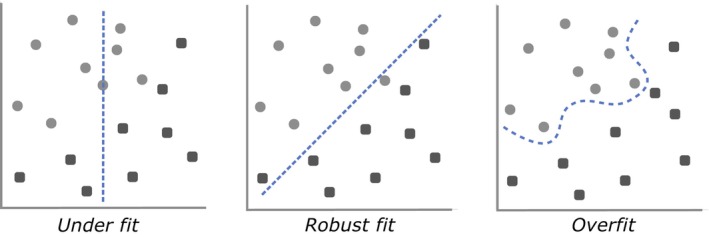
Overfitting occurs when a model is too well adjusted to the specific noise of the training data. Such models often perform deceptively well on the training data, but poorly on new data. More robust models are those that find underlying signals in the data and can generalise to new instances. Figure adapted from Montesinos López et al. (2022).

Overfitting is among the most commonly encountered, yet least‐recognised risks of ML (Chicco, 2017; Yates et al., 2023). Overfitting occurs when the model's complexity approaches or surpasses that of the data (Figure 2). This causes the model to overadapt to the context of the training set, essentially ‘memorising’ specific nuances in the training data rather than learning to recognise more generalised patterns that apply beyond the training data (Chicco, 2017; Goodfellow et al., 2016; Xu & Goodacre, 2018). Despite initially appearing highly accurate—even approaching perfect performance, on the training data—overfit models will often perform poorly on the test set, and struggle when applied to new instances, individuals or scenarios that differ from the training set (Chicco, 2017; Goodfellow et al., 2016).

Overfitting is an inherent risk in all fitting algorithms but is more common in larger models with more free parameters and especially problematic for high‐dimensional, non‐statistically based models such as deep learning neural networks (Hosseini et al., 2020). Overfitting can be prevented with various techniques, mostly aiming to intentionally limit the model's ability to memorise the training data (Chicco, 2017). To properly implement these controls, however, overfitting must first be detected.

A tell‐tale sign of overfitting is a significant drop in performance between the training set and an independent test set, indicating that the model has low generalisability to new datasets. This deterioration in performance, however, is frequently obscured by incorrect validation procedures. Common practices in ML validation that may mask overfitting include (i) a lack of independence of the testing set, (ii) non‐representative selection of the test set, (iii) failure to tune model hyperparameters on a validation set and (iv) optimisation on an inappropriate performance metric (Greener et al., 2022; Hosseini et al., 2020). Our review of validation techniques used in supervised ML, as applied to classification of animal behaviour using accelerometer data, seeks to determine the potential scope of these practices in this field and to suggest guidelines for avoiding common pitfalls in future studies.

## 2 MATERIALS AND METHODS

To explore the extent of overfitting in the animal accelerometer literature, we conducted a systematic review under the Preferred Reporting Items for Systematic reviews and Meta‐Analyses (PRISMA) standard (Page et al., 2021). The PRISMA standard was designed to aid the transparent reporting of systematic reviews, covering motivation, method and results of the systematic review in clearly defined stages (Page et al., 2021). No ethical permits were required to undertake this review.

We defined eligibility criteria as ‘peer‐reviewed primary research papers published in 2013 until present that use supervised machine learning to identify specific behaviours from raw, non‐livestock animal accelerometer data’. We elected to ignore the analysis of livestock behaviour as agricultural methods often operate within different constraints to the analyses conducted on wild animals and this body of literature has mostly developed in isolation to wild animal research. Our search was conducted on 27 September 2024. Initial keyword search across three databases (Google Scholar, PubMed and Scopus) yielded 249 unique papers. Papers outside of the search criteria—including hardware and software advances, non‐ML analysis, insufficient accelerometry application (e.g. research focused on other sensors with accelerometry providing minimal support), unsupervised methods and research limited to activity intensity or active and inactive states—were excluded, resulting in 119 papers.

Each of these selected papers was reviewed by a single reviewer to manually extract key information on validation methods. The information extracted from each of the included papers was as follows:

- Study system: Species, sample size and whether subjects were captive or free‐roaming;
- Validation methods: Data split partitions, data split method and validation technique (cross‐validation or other);
- Window settings: Overlap (as percentage);
- Tuning: Hyperparameter tuning, feature selection and model selection (e.g. window length, sampling frequency);
- Outcomes: Reported performance metrics.

Information from reviewed literature in supplementary materials (Dryad Digital Repository DOI: 10.5061/dryad.fxpnvx14d; Wilson et al., 2025).

## 3 DISCUSSION

### 3.1 Non‐independence of the test set masks overfitting to the training data

To evaluate a trained ML model's performance, labelled data must be divided into independent subsets for training and evaluation—the critical requirement being that the model is tested on data totally unseen by the model, as will be the case in real‐world application (Ferdinandy et al., 2020; Greener et al., 2022; Roberts et al., 2017). ‘Data leakage’ arises when the evaluation set has not been kept independent of the training set, allowing inadvertent incorporation of testing information into the training process. This leakage compromises the validity of the evaluation as the test data are more similar to the training data than unseen data would be. The similarity between training and test sets masks the effect of overfitting, causing an overestimation of model performance compared to true performance on unseen data (Chicco, 2017; Ferdinandy et al., 2020; Goodfellow et al., 2016). While this general concept is typically well understood by researchers, the nuance and specifics of how exactly such data leakage arises can be misunderstood.

**FIGURE 3:**
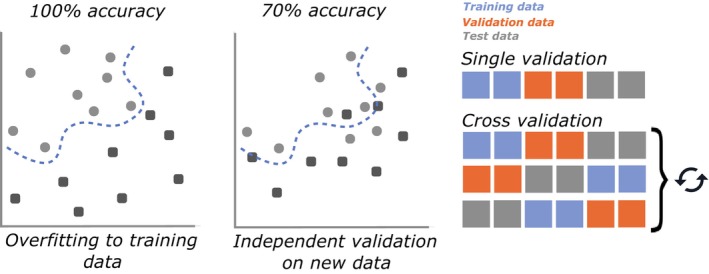
Validation on new data not included in the training set enables overfitting to be detected. Left shows a model overfit to the noise of the training data. When this model is applied to new data, the noise now provides a disadvantage, suggesting the model is overfit (middle). This validation can be single, with a single division into training, validation and testing data. Alternatively, cross‐validation shuffles and redivides the data multiple times with new portions of the same data assigned to training, validation and testing for each iteration.

Model validation largely falls into categories of singular validation and k‐fold cross‐validation (Figure 3). In singular validation, the data are split once, with the test data held in a ‘vault’ and not accessed until the final evaluation of model performance. In cross‐validation, the data are segmented *k* times (i.e. *k* folds) and evaluation is repeated for each of these folds (Yates et al., 2023). Alternatively, bootstrapping, a resampling method where samples of data are iteratively extracted to form as the test set and then returned to the sample pool before the next sample is pulled can be implemented used for estimating performance variance (Harrell, 2001; Montesinos López et al., 2022). Bootstrapping appeared infrequently in the animal accelerometer‐based behaviour classification literature but is similar to cross‐validation, except that individual samples may appear more than once, and others never appear (Montesinos López et al., 2022). For each of these methods, the same risks apply.

Typically, it is assumed that random subsampling will eliminate data leakage (Aulsebrook et al., 2024; Chicco, 2017), but this assumption does not hold true for time‐series data, such as biologging data. Because biologging data are collected in sequence, temporally adjacent measurements are not considered independent, which is especially the case when a short window (Figure 1) bisects a longer behavioural pattern, turning a single continuous sequence into multiple similar segments (Aulsebrook et al., 2024; Ferdinandy et al., 2020; Minasandra et al., 2023; Roberts et al., 2017). Random division into training and testing sets risks these related contiguous windows being split between the two datasets, epitomising the phenomenon of data leakage (Mannini et al., 2013). In this case, the cross‐contamination means that the training and test sets are correlated, which means that models overfit to the training data will have an unfair advantage when assessed against the test data, maintaining high performance on the non‐independent test set (Figure 3). As such, random subsampling artificially inflates accuracy estimates compared to true performance on independent, unseen, data. The use of overlapping windows (where adjacent windows sample from the same underlying data) further exacerbates non‐independence, leading to explicit data duplication between testing and training sets (Dehghani et al., 2019; Mannini et al., 2013). The apparent increase in accuracy associated with overlapping windows may be the result of data leakage rather than a true increase in performance (Dehghani et al., 2019).

**FIGURE 4:**
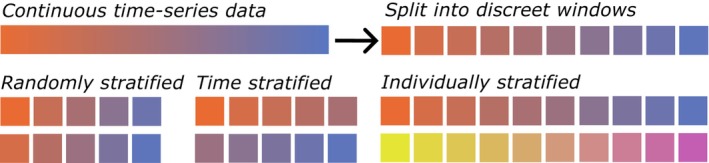
When continuous time‐series data are split into discreet ‘windows’, consecutive windows will be related to each other. When then splitting these data into training and testing sets, splitting consecutive windows into different sets can result in overly similar sets (known as ‘data leakage’) that can mask model overfitting. Randomly stratified windows can result in consecutive windows being separated to training and testing sets. Time‐stratified windows better separate consecutive windows, but retain some relation. Individual stratified sets have no shared information between the training and test sets, with the lowest risk of data leakage and overfitting.

One alternative to random subsampling is a subject‐based or leave‐one‐individual‐out (LOIO) approach, where the model is trained on the full labelled dataset from some individuals, validated on the full set of others and tested on the complete set of a single (or multiple) other individual(s), thereby ensuring total independence of the test set (Ferdinandy et al., 2020; Goodfellow et al., 2016). This method tests the performance of the model when applied to new data and individuals not contained within the training set, and it is appropriate for situations where the model will ultimately be applied to unlabelled data from new individuals, as is the ultimate aim in most instances for animal accelerometer research. Because the test data are independent from the training data, overfit models incur no advantage, and reported performance more closely mimics true performance on the unseen data (Figure 4).

Alternatively, when labelled data are available only from the same individuals as the unlabelled target data and there are too few individuals for LOIO validation, a time‐stratified method could be used to minimise the impact of overfitting (Aulsebrook et al., 2024). This involves splitting data chronologically, often using the initial portion of an individual's data for training and the subsequent portions for validation and testing, ensuring independent sequences appear in each set. Application of this method is common in other areas of time‐series analyses, such as finance and weather prediction (Nielsen, 2017). Because animal behaviour is deterministic, temporally distant instances will not be completely independent, but will be less correlated than temporally close samples, making time stratification a more appropriate choice than random subsampling when LOIO is not possible (Aulsebrook et al., 2024; Swihart & Slade, 1997).

Of the papers reviewed, 25% (30 papers) did not report sufficient information for us to determine the validation method used, 47% (56 papers) reported use of cross‐validation, 23% (28 papers) used singular validation and 2% (3 papers) made use of both types. Combining all methods, 18.5% of studies (22 papers) did not report the method used to split out the test data, 47% (56) validated solely on randomly split data, 19% (23) verified with LOIO splits and 10% (12) combined random sampling with an alternative independent validation method. Thus, nearly half of the studies drew conclusions based on randomly sampled test sets, masking potential overfitting to the training set. Trends over time indicate an increase in the proportion of papers reporting validation methods, from 60% (20 of 30) in 2013–2018 to 87% (74 of 85) in 2019–2024. However, the practice of random data splitting has become more common, being used in 40% (12) of papers published from 2013 to 2018, increasing to 50% (43) in 2019–2024. The majority (56%; 9 of 16) of papers published thus far in 2024 relied on random splitting.

### 3.2 Model selection and hyperparameter tuning on the test set masks overfitting to the test set

Hyperparameters are variables that cannot be learned by the ML model during training but are set prior to training (Yu & Hong, 2020). Examples include model‐specific settings such as the algorithm type, learning rate or ‘size’ of the model, as well as preprocessing decisions, for example, the window length, degree of overlap between windows and which features are used to develop the training data in statistical models. These decisions tailor the model to the specific learning problems it is presented with. Given the diversity in data quality, quantity and the complexity of classification tasks varying between different datasets, no single ML model will be appropriate for all contexts (Greener et al., 2022). Each ML architecture is based on unique assumptions, aligning most effectively with data that meet those assumptions, so there is no optimal set of hyperparameters, but rather a set of hyperparameters that is most appropriate for the problem at hand.

Hyperparameter tuning typically involves training models with a range of possible parameters and evaluating the performance of each model variant on an evaluation set to identify the parameters associated with best performance. These potential parameters can be identified using grid search (exhaustively trialling possible combinations), random selection (a random selection of possible options), Bayesian estimation (incrementally searching for global optima) or algorithms based on population or evolutionary dynamics (Chandrashekar & Sahin, 2014; Yu & Hong, 2020). Hyperparameter tuning typically involves selecting a set of hyperparameters as found by the search algorithm, training a model according to these parameters and then evaluating model performance on a new evaluation set. The hyperparameter combination with the best classification performance is then selected as the final model. It is critical, however, that this optimal hyperparameter performance is not mistaken for the generalised performance.

**FIGURE 5:**
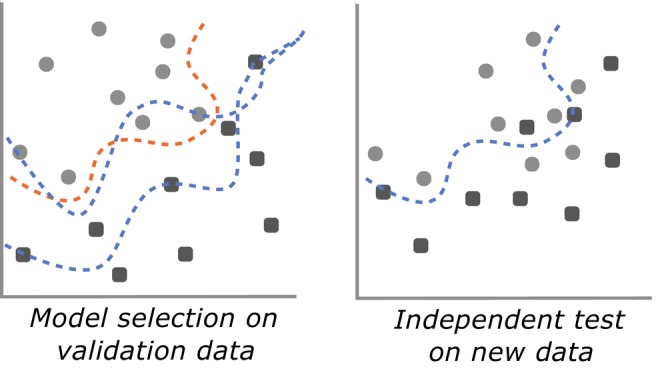
How tuning of hyperparameters can overfit to the validation set. Many potential models are generated using the training data and evaluated on the validation data. Given enough free parameters and possible iterations, by chance, one of the models may fit the evaluation data (orange line). Final evaluation using additional independent test data prevents overestimation of accuracy due to overfitting to the tuning set.

As the number of free model parameters and iterations among these parameters increases, so does the likelihood that one of the possible model parameter sets will be overfit to the evaluation data. Analogously to ‘p‐hacking’—where running enough statistical tests eventually yields a seemingly significant result—tweaking a model until evaluation reports high accuracy makes it difficult to know whether the model genuinely represents the signal or has simply overfitted to the evaluation set by chance (Figure 5; Quinn et al., 2021). To distinguish between genuine model performance and overfitting, a third independent dataset is necessary. This is known as the ‘validation’ set and is used to fine‐tune hyperparameters while safeguarding against overfitting before evaluating on the final evaluation (test) data (Cawley & Talbot, 2010).

**FIGURE 6:**
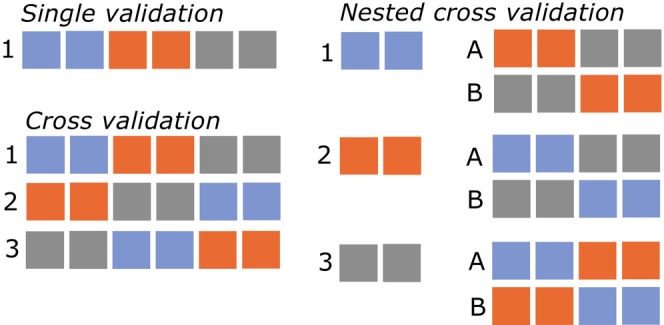
Single validation versus nested cross‐validation. In single validation, the data are divided once into training, validation and testing portions. In cross‐validation, each portion of data is iteratively assigned to the training, validation and testing roles. In nested cross‐validation, there is both an outer loop (each portion of data iteratively assigned to the testing role) as well as an inner loop (each portion of remaining data iterates between training and validation). Colours indicate partitions of data that are shuffled in each cross‐validation.

This validation set is necessary for hyperparameter tuning procedures, whether in a simple train–validation–test split or within a cross‐validation procedure. While cross‐validation (which alternates between training and testing splits) is often thought to mitigate overfitting, it does not eliminate the risk entirely when used for model selection and hyperparameter tuning (Cawley & Talbot, 2010; Hosseini et al., 2020; Yates et al., 2023). Each time a model is evaluated on a dataset, the performance provides information about that dataset. As tuning is part of the training (not testing) phase of model development, tuning by calculating performance on the test set compromises the independence of the test set—information about the test set has been used to inform the training process, which is a form of data leakage (Fannjiang et al., 2019a; Quinn et al., 2021; Xu & Goodacre, 2018). To overcome this limitation, nested cross‐validation can be used, where an inner loop tunes the hyperparameters and an outer loop evaluates the model (Figure 6; Cawley & Talbot, 2010; Hosseini et al., 2020; Yates et al., 2023). Although this repeated loop of validation is computationally expensive, this level of robust validation is necessary to adequately detect overfitting during model tuning and to prevent overfit models from incurring unfair advantage. While this method has been found to be overkill in scenarios with few tuneable parameters (Wainer & Cawley, 2021), the degree to which this may or may not be necessary for animal accelerometry remains to be investigated.

In the animal accelerometry literature, a review of the prevalence of hyperparameter tuning approaches (e.g. costs, weights, depth) found that this stage of model development was infrequently reported. Including feature selection—selecting a subset of promising features for use in model development in statistical‐based ML models (Aulsebrook et al., 2024; Demircioğlu, 2021)—and other elements of model selection, such as trialling a number of window lengths and sampling frequencies in tuning procedures, 57% (68) papers reported on model tuning. Of these, 14% (10) assessed performance on a dedicated validation set and a further 13% (9) performed inner cross‐validation within the training set; 48% (33) did not implement tuning on any kind of validation set (predominantly implementing default parameters), with a further 23% (16) not reporting the number of data portions the labelled set was split into. Use of a validation set, but one that is not independent from the training and testing data (see section above), risks masking overfitting as well, and the method of dataset division should also be considered when assessing test set independence. Of the 68 papers that reported model tuning, 10% (7) validated on data that could be considered meaningfully independent from the training and testing sets (i.e. not randomly split).

The impact of information leakage through overfitting hyperparameters to the test set is often underappreciated and frequently overlooked, even in long‐established ML research (Cawley & Talbot, 2010; Hosseini et al., 2020). While our review cannot definitively confirm overfitting to the test set, it shows that current validation protocols are insufficient to detect the phenomenon. It is nevertheless reasonable to infer that overfitting likely occurred in much of the animal accelerometry literature, as has been common in other fields during early ML adoption.

### 3.3 Inappropriate performance metrics prevent meaningful model optimisation

The performance of supervised classification models is typically evaluated using a confusion matrix, where known true categories are organised as rows and predicted categories as columns. Each cell in the matrix contains counts of observations, with the diagonal indicating correct classifications. While confusion matrices provide comprehensive insights into model performance, they can become challenging to interpret in multi‐class scenarios. Consequently, performance evaluation often relies on a more manageable set of metrics. The appropriate choice of these test metrics is critical for model validation, as it is through these metrics the performance of the model can be understood and the most appropriate model optimised for (Ferri et al., 2009). There is no universal ‘best’ metric, but rather many possible metrics, each of which reports different elements of performance from the confusion matrix (Ferri et al., 2009; Lovell et al., 2023). Selecting the appropriate set involves careful consideration of the goals of the optimisation balanced against the ‘blind spots’ of the metrics.

Accuracy is the most commonly referenced performance metric in classification tasks. Defined as the proportion of correct predictions made by a model, this metric provides valuable insights into model performance, but it is often insufficient, particularly for imbalanced datasets (Ferri et al., 2009; Sur et al., 2023). In situations characterised by class imbalance, accuracy can be inflated by models that predominantly predict the majority class (e.g. in a dataset with a high proportion of sleeping data, if 80% of instances are ‘sleeping’, a model that predicts sleeping for all instances will achieve an accuracy of 80%; Goodfellow et al., 2016). Relying solely on accuracy can obscure a model's true performance, so the inclusion of additional performance metrics is recommended.

Recall measures the proportion of correctly identified positives (true positives, TP) out of all actual positives (TP + false negatives, FN), providing insight into the model's effectiveness in capturing positive instances. Specificity measures the proportion of true negatives (TN) out of all TN + false positives (FP), reflecting the model's ability to accurately identify negative classes. Precision (TP/(TP + FP)) assesses the recognition of positive classes. While each of these metrics is more easily interpreted in the binary context (as one‐vs‐all in the multi‐class scenario), macro‐averaging the scores from each class provides multi‐class performance estimates (Kautz et al., 2017). These scores, however, are also similarly sensitive to class imbalance and, used alone, should be interpreted with caution (Kautz et al., 2017).

Compound metrics, such as the *F*1‐Score or Matthews Correlation Coefficient (MCC), are said to be more robust to class imbalance because they draw from multiple elements of the confusion matrix (Chicco, 2017). *F*1‐Score balances precision and recall by calculating their harmonic mean, providing a single metric that accounts for both false positives and false negatives (2 × Precision × Recall/(Precision + Recall)). MCC improves upon this balance by further incorporating all four categories (TP, TN, FP, FN) to provide a more holistic performance overview ((TP × TN − FP × FN)/sqrt((TP + FP)(TP + FN)(TN + FP)(TN + FN))).

All above‐mentioned metrics rely on the selection of a specific threshold, which determines the class assigned to a prediction based on the model's confidence score. In contrast, rank‐based metrics evaluate model performance across a range of thresholds (Ferri et al., 2009). Two common rank‐based metrics are the area under the receiver operating characteristic curve (AUC‐ROC) and the area under the precision‐recall curve (PR‐AUC) (Cook & Ramadas, 2020). AUC‐ROC measures the trade‐off between sensitivity and specificity at various thresholds, providing insights into the model's ability to distinguish between classes. PR‐AUC focuses on the trade‐off between precision and recall, highlighting the model's performance on the positive class, which is particularly helpful when the positive class is in the minority (Cook & Ramadas, 2020). While these methods are calculated for binary classes, they can be generalised to the multi‐class by macro‐averaging one‐vs‐all scores for each class.

In our review, we found that 74% (88) of papers reported model accuracy, with 26% (31) citing accuracy as the sole performance metric. 40% (48) of the studies reported an accuracy exceeding 90%, while 70% (83) reported accuracy above 80%. The next‐most frequently reported metrics were recall (54 papers) and precision (52), followed by specificity and F1‐score (both reported in 25 papers). Other metrics, including AUC, PR‐curve, MCC and Kappa (accuracy accounting for chance baseline; Ferri et al., 2009) each individually appeared infrequently. It is not yet clear which of these metrics or combinations of metrics are the most appropriate for validation in animal accelerometry, and until a dedicated study is undertaken, it remains up to the author to justify the metric/s they present. Such a dedicated study, either a simulation study with case‐study data or a review of metrics implemented in other fields and their advantages and trade‐offs, may be sufficient to solve this challenge. However, as currently, no definitive guidelines can be given, present best practice would be to consider a range of metrics in context of the study's goals as well as reporting the upper and lower bounds of performance between cross‐validation folds (i.e. the uncertainty) where cross‐validation has been performed (Lovell et al., 2023).

### 3.4 Unnatural test sets optimise for unnatural models

The essential goal of machine learning is to generalise beyond the training set to new, unseen data (Domingos, 2012). It is possible to report the performance of ML models against the test set, but not how appropriate the test set was for the problem. To generalise well, the test data must, as much as possible, mimic the unseen target data in terms of behavioural stratification, environment and types of individuals (Dickinson et al., 2020; Ferdinandy et al., 2020; Yu et al., 2022). It is the responsibility of the researcher to determine how similar the test set is to the real data and decide whether the calculated performance metrics are generalisable to the final application.

‘Gold‐standard’ validation would be to collect labelled training data from multiple individuals of similar status (i.e. environment, size, behaviours) to the ultimate unseen research individuals and validate the model with appropriately partitioned LOIO methods on these labelled individuals. Animal research, however, often poses logistical, practical and ethical challenges that can limit data collection, placing the ‘gold standard’ beyond reach (Lenth, 2001; Patterson et al., 2019).

Implementing hold‐out test data for validation ensures final evaluation is on truly unseen data, providing a fair estimate of true model performance, provided that the test data are drawn from the same distribution as the training data (Hastie et al., 2010). In cases of limited sample size, implementing a hold‐out test set containing only a single individual can be risky, as this single individual may introduce biases, or exhibit individual idiosyncrasies to the degree that it may render the individual unrepresentative of the population in general (Chimienti, 2022). Where there are sufficient data to provide a test set composed of multiple individuals, hold‐out data are preferable, but where such data are insufficient, LOIO cross‐validation can mitigate risks of biased test individuals. By iteratively calculating cross‐validated test performance on each of the individuals, LOIO cross‐validation provides an average performance to account for the bias of any one individual, as well as an additional metric of ‘uncertainty’ (how the performance changes among individuals) which can be used to choose between models, balancing both average performance and the performance range. The final model, built on the data from all individuals, is assumed to have performance approximately equal to the average performance from each of the folds (Hastie et al., 2010).

Capturing the full behavioural range of a species is often also impractical if not impossible (Campbell et al., 2013). Free‐roaming animals often move beyond the reach of researchers' observation, while captive animals may exhibit atypical behaviours or only a narrow range of their natural repertoire, meaning not all behaviours that occur in the unlabelled, unseen data are captured in the labelled set (Chimienti, 2022; Dickinson et al., 2020; Ladds et al., 2016). While some literature recommends the use of captive surrogates of alternate species—both close (Ferdinandy et al., 2020) and distant phylogenetic relations (Campbell et al., 2013)—other research suggests that captive surrogacy is ineffective, in some cases, even when the surrogate is from the same species (e.g. Pagano et al., 2017). In these instances, data from captive individuals may not sufficiently represent their free‐roaming counterparts due to constraints of enclosures or ethical considerations leading to unnatural movements (Dickinson et al., 2020; Pagano et al., 2017). Similarly, human biologging research consistently reports a 20–30% decrease in model performance when laboratory‐trained models are deployed on free‐roaming people (Farrahi et al., 2019). While not ‘overfitting’ in the traditional sense, this limitation nevertheless results in an optimistic performance estimate, potentially not generalising to the true performance in the wild (sample of papers compared in Table 1). Similarly, when only a limited set of behaviours are collected, or only clean examples included (i.e. ‘other’ classes removed and transitions between behaviours eliminated), a highly tailored and unnaturally simplistic dataset is developed (Resheff et al., 2024). Despite high accuracy on this curated dataset, the model's practical value diminishes when this fails to represent the true behavioural range (Resheff et al., 2024).

**TABLE 1:** Deploying captive‐trained models on free‐roaming individuals. Despite achieving high accuracy on the captive set, the ML model can display significant limitations when applied to the target free‐roaming individuals, often being unable to detect realistic free‐roaming behaviours.

Reference	Species	Model	Captive accuracy	Free‐roaming performance
Fannjiang et al. (2019b)	Jellyfish	Discriminant Analysis	0.99	Unable to detect wild behaviours when trained on only data from captive individuals. Addition of in situ free‐roaming data improved classification performance
Rast et al. (2020)	Fox	Random Forest	0.955	Unable to detect wild behaviours using Random Forest or Support Vector Machine models (all samples were classed as ‘grooming’). Able to detect multiple behaviours only using the neural network
		Support vector machine	0.8817	
		Neural network	0.9433	
Pagano et al. (2017)	Polar bear	Random forest	Not assessed	The captive‐trained model was able to detect stationary behaviours only. Only wild‐trained models were able to distinguish energetic behaviours
Clarke et al. (2021)	Pelagic fish	Random forest	0.94	‘Swimming’ was not detected in 3 of 5 free‐roaming individuals (likely due to large fish size increasing signal magnitude)
Harvey‐Carroll et al. (2024)	Pangolin	Random forest	0.85	Observed to generate reasonable free‐roaming behavioural budgets
Dunford et al. (2024)	Cat	Random forest	*F* = 0.96 (accuracy not reported)	Despite achieving high test ‘accuracy’, some models failed to identify grooming and feeding in free‐roaming cats. Other models were able to identify these behaviours

Although the accuracy of models could not be assessed for free‐roaming individuals due to the absence of ground‐truthed data, models trained on captive specimens often failed to reliably detect free‐roaming behaviours. To expand the use of accelerometry in wild populations, the development of effective methods for transferring captive‐trained models to free‐roaming individuals is a priority.

### 3.5 Uncertain reporting obscures methods

A limitation of this systematic review was the inability to automate key data extraction due to inconsistent reporting across studies, a well‐recognised and long‐standing issue in this field (Brown et al., 2013; Campbell et al., 2013). For instance, the well‐established ML term ‘windows’ was inconsistently referred to as segments, increments, periods or epochs—the latter having an alternative specific meaning in ML as ‘data presentations’ for training neural networks (Goodfellow et al., 2016). The overlap between windows was often vaguely described with terms like ‘rolling’, ‘sliding’ or ‘moving’, and even the term ‘cross‐validation’ was sometimes used ambiguously, making it unclear whether it referred to a single or multiple validation folds. This inconsistency also hampered the qualitative assessment of missing information, making it unclear whether omissions, such as the absence of hyperparameter tuning details, were intentional null values or simply incompletely reported.

We informally observed that, compared to details on the study system (e.g. species, sample size and data collection methods), validation methods were reported less thoroughly. For instance, 18% of papers (22) lacked sufficient information to determine the method of data splitting. In 38% (45) of papers, the portions of data used were unspecified, making it unclear whether a validation set was included. In 25% of papers (30), it was not possible to determine whether cross‐validation or single validation had been used.

The implementation of a standardised reporting checklist for animal accelerometry ML studies would greatly enhance reproducibility and compatibility in this field. The Data Optimisation Model Evaluation (DOME, Walsh et al., 2021) guideline is a field‐agnostic, generalised, biology‐accessible checklist suggested for reporting supervised ML analysis. While not intended to be exhaustive, adhering to this checklist could assist future biologging studies to ensure transparency and reproducibility, facilitating robust scientific advancements.

### 3.6 Best practices for detecting overfitting

In light of these common challenges with machine learning validation, the following concepts should be considered to ensure best practice for detecting model overfitting:

1. Non‐independence of the testing set masks overfitting to the training data.

Labelled data should be split such that the testing data are independent from the training data. With a sufficient sample size, data can be split into three independent subsets (training, validating and testing), or, when there are insufficient data, nested cross‐validation should be considered. For time‐series data, random subsampling within individuals should be avoided, and LOIO or chronological splits should be used instead. Exemplary discussion of testing set independence is demonstrated (Aulsebrook et al., 2024; Ferdinandy et al., 2020).

2 Hyperparameter tuning and model selection on the test set masks overfitting to the test set.

Tuning hyperparameters is an important stage of fine‐tuning models to specific traits in the dataset. This process must be completed as part of the training process prior to evaluation of the final model on the test set. With sufficient data, this will require three separate and independent subsets of data and, for cross‐validation workflows, a nested cross‐validation (with an inner training/validation cross‐validation and then an outer test cross‐validation) may be implemented. An exemplary discussion of the importance of hyperparameter tuning is found in Hosseini et al. (2020).

3 Inappropriate performance metrics prevent meaningful model optimisation.

Performance metrics are critical for guiding the optimisation and selection of models. Selecting a limited set of performance metrics from a complete confusion matrix is necessarily reductive and each metric presents its own limitations and biases. Particularly in the context of class imbalance, be wary of accuracy as a stand‐alone metric and consider multiple metrics and compound metrics in evaluations. When using cross‐validation, report the full range of performance variation across validation folds. Exemplary discussion of performance metrics can be found (Ferri et al., 2009).

4 Unnatural test sets optimise for unnatural models.

Ensure collected data capture real‐world variability by including a broad spectrum of individuals, behaviours and transitions, reflecting real‐world model application as much as possible. Model predictions are directly applicable only to the subpopulation, behaviours and context contained within the labelled data. Extrapolating beyond these constraints, or use of surrogates, should be carefully justified, and results caveated and interpreted with caution. Exemplary discussion of limits to generalisation can be found (Dickinson et al., 2021; Ladds et al., 2016).

5 Adherence to the DOME reporting guidelines ensures reproducible ML.

Standardising reporting of methods across the literature by use of the DOME guidelines (Walsh et al., 2021) for supervised ML would ensure that future research can more easily learn from research in the past. For validating accelerometer‐based animal behaviour classification models, specifically, the following should be clearly and explicitly stated:

- Method of splitting (random, chronological, stratified, by individual, other—with justification);
- Portions of splitting (training/validation/testing, with proportions);
- Method of validation (single, cross‐validation, inner cross‐validation with hold‐out test set, other);
- Performance metrics (a range of metrics—with justification—as well as performance range across folds);

6 Sanity checks and control conditions in the ML workflow help to avoid errors.

Similar to the use of ecological baselines or null conditions in other ecological models, an ML control could train and evaluate the model on a randomised dataset, where poor performance would be expected. This would confirm that the model is responding to real conditions rather than erroneous code or leakage. Critically, we encourage this developing research field to see ML as more than a tool, but an experiment in its own right. ML can be an incredibly powerful tool for pattern recognition, but the onus is still on the ecologist to mistrust the results until rigour is proven, critically evaluating whether the reported results—particularly when performance is reported to be very high—are generalisable and trustworthy.

## 4 CONCLUSION

Combining the 18 papers that used independent training and testing sets without model tuning and the seven papers that tuned models using independent validation sets, this review found that only 25 papers (21%) in the reviewed animal accelerometer‐based behaviour classification literature followed ‘gold standard’ ML validation methods. The remaining 79% of the literature (94 papers) did not validate their models in a way that could reveal overfitting. Despite 70% of the studies reporting model accuracy above 80%, our review suggests that inconsistent validation practices may be concealing overfitting in many, if not most, of these studies.

Literature review alone cannot determine the actual impact of this potential overfitting, as suboptimal validation does not inherently mean that a model is overfit or that performance has been overstated. However, without gold standard validation, it is impossible to determine whether overfitting or accuracy inflation occurred, leaving the results uncertain. In these situations, both the capacity of a model to generalise and the ecological conclusions must be treated with caution. An example of the potential effect of masked overfitting is available in a re‐evaluation of past work by the present authors using Supervised Self‐Organising Maps to classify behaviours in various species (Annett et al., 2024; Galea et al., 2021; Gaschk et al., 2023). These studies used random data splitting for training and testing data, did not implement a validation set for hyperparameter tuning and prioritised accuracy in imbalanced class scenarios. Although each paper reported 99% classification accuracy, our re‐analysis accounting for the aforementioned limitations demonstrated that generalised model performance on an independent test set was actually only around 50%, indicating substantial overfitting in the original papers. While this is only one example of the impact masked overfitting can have on the predictive power of behaviour classification models, it is possible that a similar impact could be hidden across the literature.

Overfitting is a persistent challenge across most ML implementations to which ecology is no exception (Ginzburg & Jensen, 2004; Roberts et al., 2017). For instance, camera trap species identification models trained and tested on images from the same camera location and time of day can become overfitted to specific image backgrounds and must be tested across a range of contexts before deployment (Norouzzadeh et al., 2021). Similarly, acoustic detectors can achieve high performance when trained and tested on samples from the same audio file but may fail to generalise beyond these scenarios; their true performance must be validated across a range of independent recordings (Kershenbaum et al., 2025). Even non‐ML‐based plant biomass estimation models have been shown to produce overly optimistic predictions when validated on spatially correlated test data (Ploton et al., 2020; Yu et al., 2021). Thus, the guidelines presented in this paper, while developed in the context of animal accelerometer‐based behaviour classification, are relevant to the development of all predictive models across ecology, particularly those implementing time series and spatially correlated analyses.

Overfitting detection and prevention is a large, complex and rapidly evolving field, with best practices being continually refined. As ecologists increasingly adopt ML into applied research, our protocols and implementations similarly advance. Although a ‘one‐size‐fits‐all’ validation method suitable for all ML applications is not possible, the fundamental principles outlined in this paper are broadly applicable across ecology. Ecologists considering implementing ML—or indeed any predictive model—in their research should carefully consider and account for how biased validation practices may limit the generalisability of their model results and remain vigilant to the possibility of model overfitting.

## AUTHOR CONTRIBUTIONS

Oakleigh Wilson conceived the idea for the paper, collected and analysed the review results and wrote the first draft. David Schoeman, Andrew Bradley and Christofer Clemente contributed ideas throughout the project and provided multiple rounds of review for the manuscript. All authors gave final approval for submission.

## CONFLICT OF INTEREST STATEMENT

The authors have no conflicts of interest to declare.

## STATEMENT ON INCLUSION

Our study was a global systematic review and was based on a meta‐analysis of secondary data rather than primary data. As such, there was no local data collection. Data were collected systematically without geographical considerations.

## Data Availability

Data available from the Dryad Digital Repository: https://doi.org/10.5061/dryad.fxpnvx14d (Wilson et al., 2025).
